# Supplementary material for: Proper experimental design requires randomization/balancing of molecular ecology experiments
Source: Ecol Evol. 2018 Jan 10;8(3):1786–93. doi: 10.1002/ece3.3687 (PMC5792580; doi:10.1002/ece3.3687)
Supplement: Supplementary file 1 [file ECE3-8-1786-s001.docx]

**Appendix 1**. Molecular ecology studies that report randomization in some part of the work. All relevant articles were screened in a randomly selected issue of four journals for the search term “random”. We deemed articles relevant when they used DNA or RNA methods that are sensitive to laboratory biases (microsatellite genotyping, SNP assays, metabarcoding, metagenomics, (meta)transcriptome comparisons, etc.). We also included also studies that use single genes for molecular identification of species or populations (e.g. barcoding and single-gene biogeographies) since identification may be non-randomly confounded by cross-contamination (a simple example would be cross-contamination when neighboring populations or related species are processed in batches). Randomization in data analysis refers to the use of mixed effect models, the generation of null hypothesis by random data rearrangements, etc.

|  | Mol. Ecol. | ISME J. | Soil Biol. Biochem. | Ecol. Evol. | J. Biogeogr. |
| --- | --- | --- | --- | --- | --- |
| Issue | 22 | 2 | 1 | 9 | 11 |
| Total relevant | 14 | 16 | 8 | 9 | 12 |
| Report randomization in sampling or data analysis | 12 | 4 | 4 | 2 | 6 |
| Report randomization in lab | 1 | 0 | 0 | 1 | 0 |
